# Exome sequencing identifies novel and known mutations in families with intellectual disability

**DOI:** 10.1186/s12920-021-01066-y

**Published:** 2021-08-27

**Authors:** Memoona Rasheed, Valeed Khan, Ricardo Harripaul, Maimoona Siddiqui, Madiha Amin Malik, Zahid Ullah, Muhammad Zahid, John B. Vincent, Muhammad Ansar

**Affiliations:** 1 Department of Biochemistry, Faculty of Biological Sciences, Quaid-I-Azam University, Islamabad, 45320 Pakistan; 2 Molecular Neuropsychiatry and Development (MiND) Lab, Campbell Family Mental Health Research Institute, Centre for Addiction and Mental Health, Toronto, ON M5T 1R8 Canada; 3 Institute of Medical Science, University of Toronto, Toronto, ON M5S 1A8 Canada; 4 Division of Neurology, Shifa College of Medicine, H-8/1, Islamabad, Pakistan; 5 Department of Psychiatry, University of Toronto, Toronto, ON M5T 1R8 Canada

**Keywords:** Intellectual disability, Mutation, Autosomal recessive, Consanguinity

## Abstract

**Background:**

Intellectual disability (ID) is a phenotypically and genetically heterogeneous disorder.

**Methods:**

In this study, genome wide SNP microarray and whole exome sequencing are used for the variant identification in eight Pakistani families with ID. Beside ID, most of the affected individuals had speech delay, facial dysmorphism and impaired cognitive abilities. Repetitive behavior was observed in MRID143, while seizures were reported in affected individuals belonging to MRID137 and MRID175.

**Results:**

In two families (MRID137b and MRID175), we identified variants in the genes *CCS* and *ELFN1*, which have not previously been reported to cause ID. In four families, variants were identified in *ARX*, *C5orf42*, *GNE* and *METTL4*. A copy number variation (CNV) was identified in *IL1RAPL1* gene in MRID165.

**Conclusion:**

These findings expand the existing knowledge of variants and genes implicated in autosomal recessive and X linked ID.

**Supplementary Information:**

The online version contains supplementary material available at 10.1186/s12920-021-01066-y.

## Introduction

Intellectual disability (ID), is a genetically heterogeneous neurodevelopmental disorder. ID is characterized by the marked reduction in individual’s intellectual capacity which is reflected in the form of intelligence quotient less than 70, and defects in adaptive behavior with an early age of onset [1]. The worldwide prevalence of ID is 1–3%, with more affected males than females [2, 3]. ID frequently co-exists with other conditions like autism, epilepsy, schizophrenia, attention deficit hyperactivity disorder or depression [4–6].

The earlier research on genetic causes of ID focused on X-linked intellectual disability and it helped in the identification of more than 100 disease causing genes. X-linked ID accounts for 10% of male ID cases which indicates involvement of autosomal gene defects in majority of the cases [7]. Additionally, copy number variants (CNVs) have been identified in patients/families with ID [8]. Next-generation sequencing (NGS) technology has created a paradigm shift in the genetic diagnosis of common and rare diseases. Application of NGS also led to a dramatic increase in disease gene identification in familial as well as sporadic ID cases [9, 10]. More than 700 genes have been discovered so far, in which a mutation can either cause ID or ID associated disorders [9, 11–14]. The present study aims to identify the pathogenic mutations in eight Pakistani families with ID.

## Material and methods

### Family recruitment and DNA extraction

The study was approved by the Bio Ethics Committee (BEC) of Quaid-i-Azam University, Islamabad, Pakistan and the institutional research ethics board of Centre for Addiction and Mental Health, Toronto, Canada. Consanguineous families with two or more ID patients were ascertained from various regions of Pakistan. Medical history was taken, and pedigrees were drawn after interviewing parents of affected individuals. Peripheral blood was withdrawn after taking an informed written consent from the respective parents of the affected individuals. DNA was extracted by using standard phenol–chloroform method.

### Homozygosity mapping and CNV analysis

Genome wide SNP microarray was performed on all available affected and normal individuals of the eight families (n = 41) by using Illumina’s Infinium Human CoreExome-24v1.3 kit (551,004 fixed markers, including ~ 284,000 SNPs (~ 1 marker every 6 Kb)), according to the manufacturer’s protocol and the Illumina GenomeStudio platform (Illumina CoreExome) was used for data processing. The SNP data was exported into PLINK format for analysis with HomozygosityMapper [15] to identify homozygous by descent (HBD) regions. For each HBD region, genotype tables were checked to confirm homozygosity and haploidentity. False positive data was excluded from downstream analysis. In case of X-Chromosome, HBD regions were identified by manual curation on genotype data and regions spanning more than 1 Mb were selected. CNV analysis was performed by using Illumina GenomeStudio cnvPartition plugin to identify likely pathogenic homozygous or heterozygous CNVs. For validation of CNV, qPCR was performed by using primers designed from within the CNV and its flanking region. Breakpoints were identified by designing overlapping primers from the flanking regions of CNV and the amplified DNA fragment was subjected to Sanger sequencing.

### Whole exome sequencing and variant prioritization

Whole exome sequencing (WES) was performed in The Centre of Applied Genomics (TCAG), Canada sequencing facility. A single ID patient from each family was used for WES, except for families MRID126, MRID137 and MRID170, for which two affected individuals were selected per family due to possible intra-familial heterogeneity. Paired-end WES was performed on NovaSeq 6000 platform (Illumina, San Diego, CA, USA) using SureSelect^XT2^ Target enrichment System (Agilent Technologies, Santa Clara, CA, USA). The sequencing data alignment, variant calling and annotation were done by using a previously described pipeline [12]. Golden Helix genome browser was used to check coverage of all coding sequences within HBD regions mapped in our families. Variant prioritization was done based on allele frequency < 0.01 in public databases, such as Genome Aggregation Database (gnomAD). Non-synonymous variants and indels in exonic and splice region were selected and further filtered based on the pathogenicity prediction by various mutation prediction tools. Variants falling either in the HBD regions or in known ID causing/associated genes were given priority. Co-segregation studies were performed for potential variants in parents and phenotypically normal members of the family by Sanger sequencing.

## Results

**Fig. 1:**
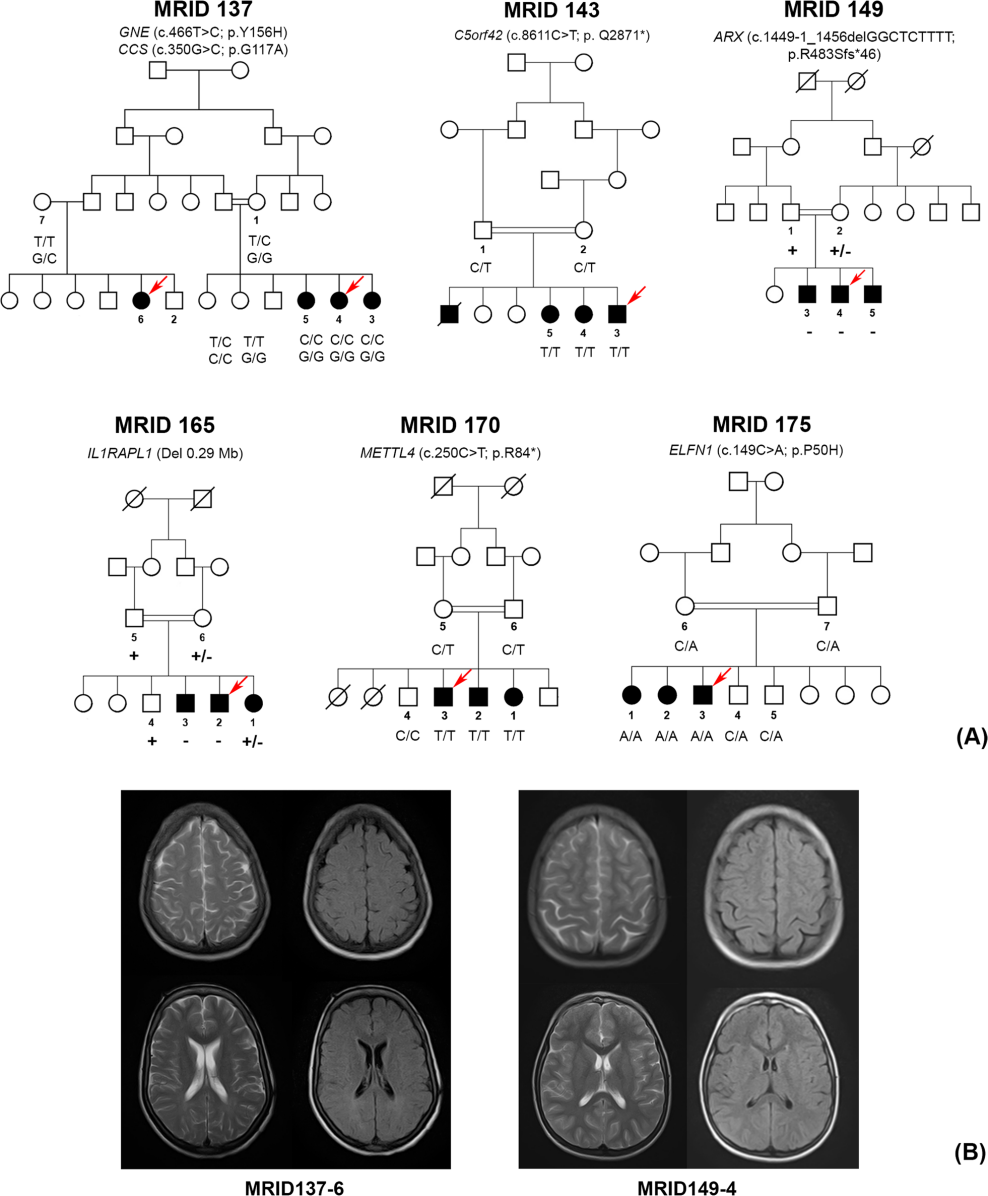
Pedigrees and haplotypes of Pakistani families (**A**). Segregation results of the identified variants are shown under the symbols of individuals available for this study. The arrow represents individuals selected for WES. Selected MRI images of patients MRID137-6 and MR149-4 (**B**)

Twenty-two ID patients, belonging to eight families, were recruited from different provinces of Pakistan. Pedigree analysis suggested the possible involvement of autosomal recessive and X linked ID in five and two families, respectively (Fig. 1; Table 1). The ages of patients ranged from 8 to 23 years at the time of recruitment and they mostly exhibited moderate to severe ID with or without dysmorphic features (Table 1), except three members of MRID143 family who presented with mild ID and bilateral strabismus. Seizures were experienced by all affected members of family MRID137 and just one affected member (MRID175-2) of family MRID175. In addition, ID patients from MRID137, MRID143 and MRID170 presented with some degree of facial dysmorphism (Table 1). A female individual MRID137-6 has muscular hypotonia, but complete blood tests revealed normal creatine kinase (CK) level (below the reference range of 167 U/L) and microcystic hypochromic anemia. Three affected male individuals of MRID149 family have testicular dysgenesis but external genitals were of normal size.

**Table 1:** Clinical features of the families

S. no.	Family ID	Individual ID	Gender	Age (years)	Head circumference (cm; SD)	Height (in.; SD)	Weight (kg; SD)	Inheritance	ID severity	Clinical features
1	MRID126	*3*	Female	21	52 − 2.17	62.4 − 0.73	66 + 0.53	AR	Moderate	Prominent jaw, Long face
		*4*	Male	19	58.5 + 2.37	68.4 − 0.40	54.5 − 1.72			
2	MRID137	3	Female	8	54 + 1.81	44.4 − 2.64	18.5 − 2.17	AR	Severe	Speech impairment, Low set ears (MRID136-6), Hypertelorism, Prominent forehead, Esotropia (MRID137-5), Facial asymmetry (MRID137-4), Seizures
		*4*	Female	15	53 − 1.07	58.8 − 1.93	40 − 1.79			
		5	Female	12	53 ± 0.0	55.2 − 1.46	32.5 − 1.46			
		*6*	Female	8	54.5 + 2.18	45.6 − 2.10	19.5 − 1.87			
3	MRID143	*3*	Male	23	59 + 2.72	69.6 + 0.03	87 + 1.12	AR	Mild	Repetitive behavior, drooling, Aggressive behavior, Strabismus, Hypertelorism (MRID143-3), anteverted nostrils (MRID143-3)
		4	Female	20	56 + 1.52	63 − 0.5	55 − 0.34			
		5	Female	18	56 + 1.52	63 − 0.44	74 + 1.11			
4	MRID149	3	Male	8	52 − 0.22	50.4 + 0.03	25.5 − 0.04	XL	Severe	Speech impairment, Impaired analytical ability, testicular dysgenesis
		*4*	Male	13	53 − 0.80	62.4 + 0.31	46 + 0.04			
		5	Male	10	52.5 − 0.23	54 − 0.22	30 − 0.46			
5	MRID165	1	Female	11	50.5 − 1.71	56.4 − 0.09	33 − 0.75	XL	Moderate	Speech unclear, Impaired analytical ability
		*2*	Male	17	52 − 2.08	61.2 − 2.30	53 − 1.18			
		3	Male	19	52.5 − 1.75	64.8 − 1.67	62 − 0.83			
6	MRID170	1	Female	21	56 + 1.52	62.4 − 0.73	63 + 0.35	AR	Severe	Facial asymmetry, Broad nasal tip and Hypertelorism in MRID170-2
		2	Male	11	–	–	–			
		*3*	Male	18	54 − 0.74	60 − 3.02	60 − 0.71			
7	MRID175	1	Female	8	48 − 2.94	46.8 − 1.55	20.5 − 1.56	AR	Moderate	Speech unclear, drooling, Seizures (MRID175-2)
		2	Female	16	52.5 − 1.69	57.6 − 2.45	42 − 1.70			
		*3*	Male	13	50 − 2.82	63.6 + 0.70	50.5 + 0.38			

DNA sample from individuals in italic type were used for exome sequencing

AR = autosomal recessive, XL = X-linked; SD = standard deviation

Brain imaging (Without contrast) from a female individual MRID137-6 (Fig. 1B) and a male individual MRID149-4 (Fig. 1B) revealed the presence of mildly prominent sulci in the parietal regions. Whereas, subtle hyperintense lesions in periventricular white matter were only noted in MRID137-6 individual (Fig. 1B). In both patients, ventricles are normal in size and are in accordance with the patient’s age.

Genome wide SNP genotyping and homozygosity mapping identified 23 HBD regions in eight families (Table 2). In MRID137, analysis of genotype data of all available family members could not detect a single homozygous region over 1 Mb in size. Further analysis of the genotype data obtained from all available members of MRID137 family detected the presence of intrafamilial heterogeneity, as members were haploidentical within the respective loop but otherwise between the two loops (MRID137a and MRID137b). But reanalysis, after splitting the pedigree into two loops (MRID137a: 1,3,4,5; MRID137b: 2,6,7) identified two HBD regions each in loop (Table 2).

**Table 2:** Homozygous by descent regions of the families

S. no.	Family ID	HBD region (hg19)	SNP ID	Cytoband	Size (Mb)	Candidate gene
1	MRID126	Chr2: 83,570,115–84,550,988	rs11894798–rs6715601	p11.2	0.9	
		Chr14: 43,433,535–44,634,641	rs1756339–rs12432423	q21.1–q21.2	1.2	
2	MRID137a	Chr9: 33,345,616–44,866,028	rs706134–rs6606438	p13.3–p11.2	11.5	GNE
		Chr9: 68,170,421–72,174,392	rs10120296–rs1970017	q13–q21.11	4	
3	MRID137b	Chr11: 64,333,296–68,033,925	rs1783811–rs7342161	q13.1–q13.2	3.7	CCS
		Chr14: 84,223,764–88,646,827	rs1958453–rs1631092	q31.2–q31.3	4.4	
4	MRID143	Chr5:34,043,649–68,044,120	rs890948–rs6898649	p13.2–q13.1	34	NNT, C5orf42
		Chr12: 67,044,058–68,434,014	rs1871549–rs721793	q14.3–q15	1.3	
5	MRID149	Chr6: 42,091,645–44,568,740	rs9471773–rs618617	p21.1	2.4	
		Chr7: 144,429,379–148,195,384	rs228587–rs749609	q35–q36.1	3.7	
		ChrX: 13,849,695–31,782,780	rs7066605–rs16998223	p22.2–p21.1	17.9	ARX
		ChrX: 124,102,283–154,916,845	rs2356746–rs669237	q25–q28	30.8	
6	MRID165	Chr2: 143,230,267–149,936,767	rs4550612–rs2377510	q22.2–q23.2	6.7	
		Chr3: 115,484,045–125,881,122	rs11929078–rs4679208	q13.31–q21.3	10.3	
		Chr4: 182,591,864–186,893,350	rs13144599–rs830829	q34.3–q4-35.1	4.3	
		ChrX: 21,213,147–42,759,963	rs9645473–rs205820	p22.12–p11.3	21.5	IL1RAPL1
7	MRID170	Chr1: 187,967,161–192,598,635	rs7543364–rs7553821	q31.1–q31.2	4.6	
		Chr7: 17,110,253–25,112,709	rs819367–rs12537364	p21.1–p15.3	8	
		Chr13: 24,430,875–27,392,427	rs7325694–rs2149132	q12.12–q12.13	2.96	
		Chr18: 2,168,420–4,312,630	rs11659972–rs3850787	p11.32–p11.31	2.14	METTL4
8	MRID175	Chr4: 166,882,179–175,964,181	rs7696087–rs1842178	q32.3–q34.1	9.08	
		Chr7: 44,935–22,233,813	rs7456436–rs3813383	p22.3–p15.3	22.1	ABCB5, ELFN1, C7orf50
		Chr4: 4,339,626–6,427,757	rs6833372–rs4469149	p16.3–p16.1	2.08	

Later WES was applied to identify pathogenic variants in the eight ID families which led to the identification of novel and known variants in seven of the families (Table 3). The identified variants were also present in the HBD regions mapped in the respective families. However, no pathogenic variant was identified in MRID126 family despite carrying out WES of two (MRID126-3 and MRID126-4) affected individuals. CNV analysis did not identify any likely pathogenic gains or losses in this family.

**Table 3:** List of mutations identified in ID families

Family ID	MRID137a	MRID137b	MRID143	MRID149	MRID165	MRID170	MRID175
Gene	*GNE*	*CCS*	*C5orf42*	*ARX*	*IL1RAPL1*	*METTL4*	*ELFN1*
Genomic Change (hg19)	Chr9:36246178A>G	Chr11:66367029G>C	Chr5:37138841G>A	ChrX:25023020delAAAAGAGCC	ChrX: ~ 0.2 Mb del	Chr18:2566966G>A	Chr7:1784381C>A
Transcript ID	NM_001190383.3	NM_005125.2	NM_023073.4		NM_014271.4	NM_022840.5	NM_001128636.4
cDNA Change	c.466T>C	c.350G>C	c.8611C>T	c.1449-1_1456del	–	c.250C>T	c.149C>A
Amino acid change	p.(Tyr156His)	p.(Gly117Ala)	p.(Gln2871*)	p.(Arg483Serfs*46)	Δex6	p.(Arg84*)	p.(Pro50His)
gnomAD Exome	7.96E−06	3.19E−04	0	0	–	4.4E−05	0
gnomAD Genome	0	3.19E−05	0	0	–	0	0
gnomAD (South Asian)	6.53E−05	2.6E−03	0	0	–	2.3E−04	0
Bioinformatics tools showing damaging results	SI, LRT, MT, Fa, PR, mSVM, mLR, M-CAP		MT, Fa	MT	–	MT	SI, MT, PR, M-CAP, Fa, MA (M)

Fa = FATHMM, LRT = likelihood ratio test, MA = MutationAssessor, mLR = meta-logistic regression, mSVM = meta-support vector machine, MT = MutationTAster, PR = PROVEAN, SI = SIFT

WES of one affected individual from the two separated loops of MRID137 identified a recurrent missense variant in *GNE* (c.466T>C; p.(Tyr156His)), in MRID137a, and a homozygous missense variant, c.350G>C; p.(Gly117Ala), in *CCS* in MRID137b. In the case of MRID143, WES data analysis revealed a novel homozygous non-sense variant, c.8611C>T; p.(Gln2871*), in exon 42 of *C5orf42*, which is known to cause Joubert syndrome and Oral-facial-digital syndrome VI [16, 17].

**Fig. 2:**
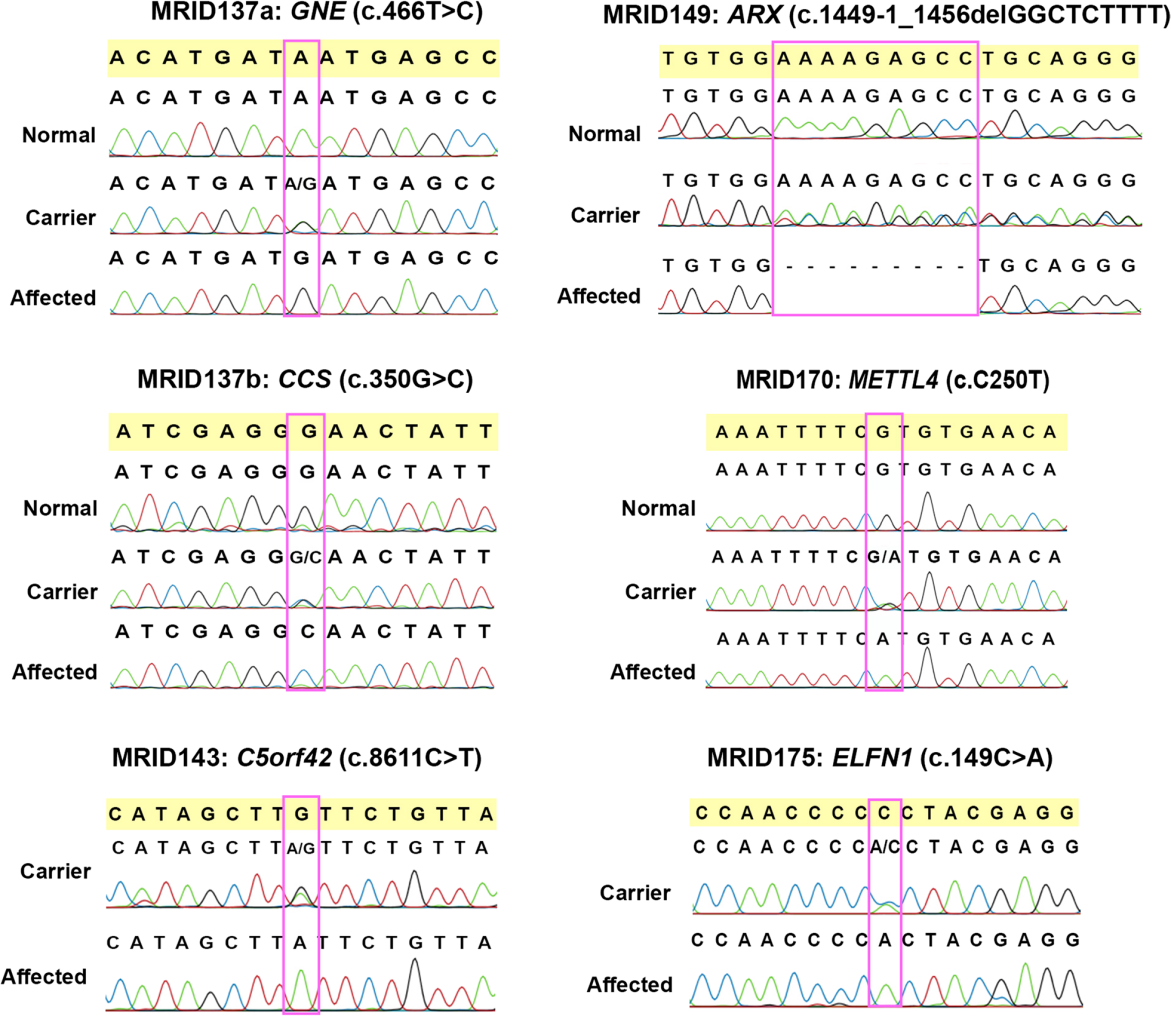
Co-segregation analysis of the variants. Electropherograms of normal, carrier and affected individuals are shown

**Fig. 3:**
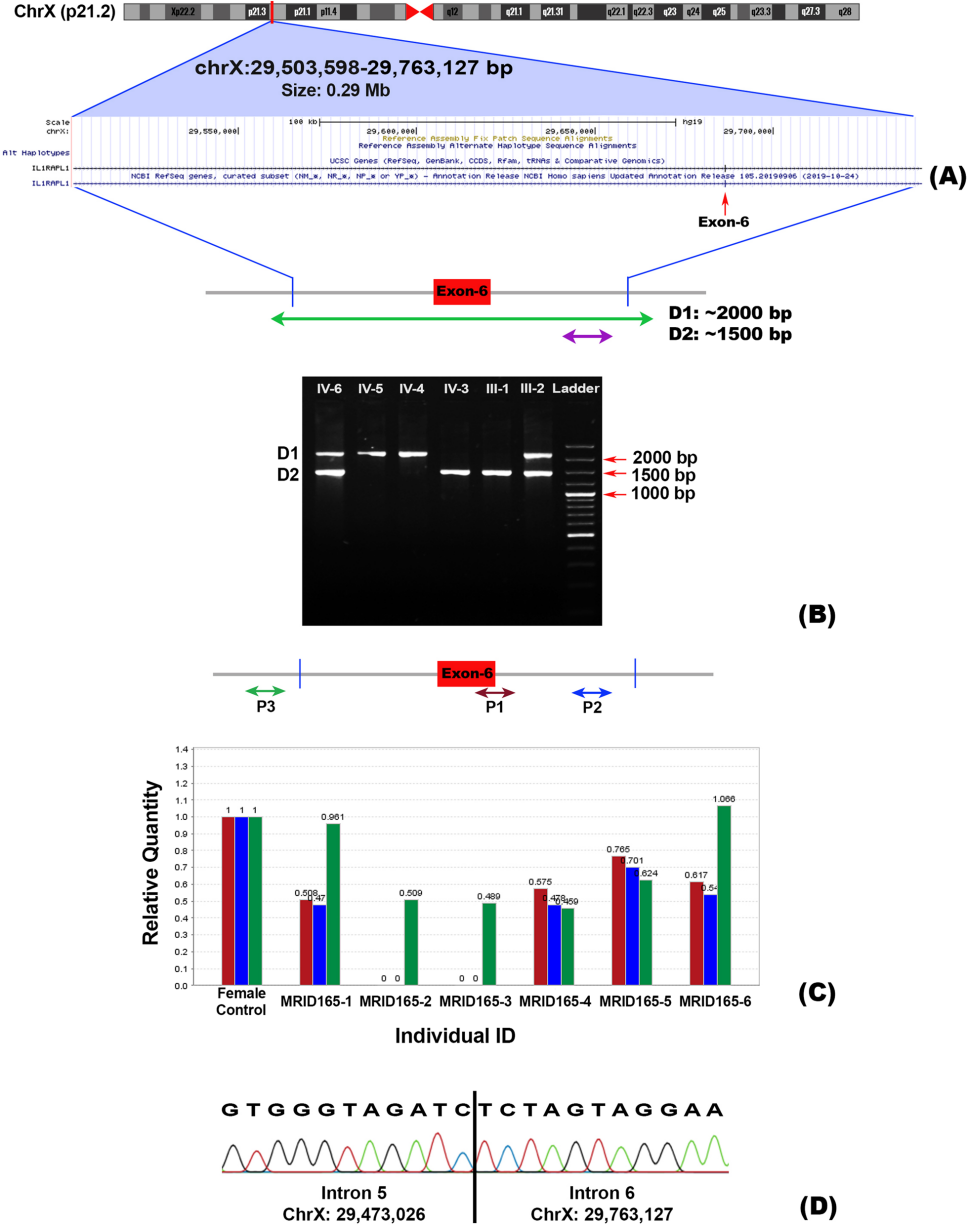
IL1RAPL1 deletion mapping performed in MRID165 family. **A** The cytogenetic location of deletion and deleted part of the gene is shown to highlight the strategy used for the confirmation of deletion. **B** Agarose gel shows the bands of products obtained with primers designed from deleted (above 2 kb) and intact (1.5 kb) regions of gene. Two affected male individuals MRID165-2 and MRID165-3 show a single band with primers designed from the deletion flanking region, but two healthy male individuals (MRID165-4, MRID165-5) show a single band of 1.5 kb with primers designed from the deleted region. Affected female individual MRID165-1 and her mother (MRID165-6) had both bands indicating heterozygous deletion on X chromosome. **C** Quantitative real time PCR analysis of IL1RAPL1 deleted region with multiple primers also confirmed the agarose gel findings. **D** Sanger sequencing results showing genomic co-ordinates (hg19) of the deleted region

In two families (MRID149 and MRID165), deletions were identified in the genes located on the X chromosome. Two homozygous regions of 17.9 Mb and 30.8 Mb were detected on X-Chromosome in MRID149 family (Table 2). WES identified a homozygous 9 bp deletion (c.1449-1_1456delGGCTCTTTT) in *ARX* which is predicted to disrupt the splice acceptor site and removes first 8 bp of terminal exon, exon 5 (Fig. 2). This is predicted to result in a frameshift mutation with premature termination of protein synthesis (p.(Arg483Serfs*46)). In the second X linked family (MRID165), an approximately 0.3 Mb deletion (Fig. 3A) was identified, which was also present within the mapped HBD region of 21.5 Mb (Table 2). This genomic deletion was initially detected by cnvPartition and was then confirmed by PCR using two sets of primers located within and around the deleted region, respectively. The presence of a single band (greater than 2 kb) on agarose gel confirmed homozygous deletion in two affected male individuals (MRID165-2 and MRID165-3), but two healthy male individuals (MRID165-4 and MRID165-5) showed a single band of 1500 bp (Fig. 3B; Additional file 1: Fig. S1). However, a female affected individual (MRID165-1) and her healthy mother (MRID165-6) have two bands indicating the presence of the deletion on a single X chromosome (Fig. 3). Real time PCR also confirmed that MRID165-2 and MRID165-3 are hemizygous for the deletion while MRID165-1 is heterozygous (Fig. 3C). Sanger sequencing of deletion flanking region from an affected male individual (MRID165-2) identified a 0.29 Mb (hg19: chrX: 29,473,026–29,763,127) deletion which spans exon 6 of *IL1RAPL1* gene (Fig. 3D). The deleted region is flanked by GATC repeat on both sides (Fig. 3D). *IL1RAPL1* deletion is expected to remove exon 6 which encodes a 25 amino acid sequence in the extracellular domain of IL1RAPL1 protein.

In MRID170, a novel homozygous non-sense variant, c.250C>T (p.Arg84*) was identified in a known ID gene, *METTL4* (Fig. 2). In MRID175 family, three potential variants c.149C>A (p.(Pro50His), c.1172T>A (p.(Val391Asp)) and c.535delC (p. Leu179Cysfs*136) were identified in *ELFN1*, *ABCB5* and *C7orf50* genes, respectively (Fig. 2). These three variants were present in the homozygous state and lie in a 22.1 Mb HBD region mapped on chromosome 7 (Table 2). The exploration of Allen brain atlas revealed the expression of *ELFN1* and *C7orf50* in different regions of brain. The c.149C>A change in *ELFN1* resulted in the substitution of an evolutionarily conserved amino acid in a region upstream to Leucine rich repeat (LRR). The functional data available for ELFN1 suggests its role in synaptic transmission by trans regulation of mGLUR7, whose mutations are already known to cause ASD, ID, microcephaly, hypotonia and seizures [18–21].

## Discussion

Next generation sequencing technology has revolutionized the process of disease gene discovery in intellectual disability. Here, we report eight families with ID and we used homozygosity mapping and whole exome sequencing to identify plausible novel disease causing mutations in *ELFN1* (p.(Pro50His)), *CCS* (p.(Gly117Ala)), *C5orf42* (p.(Gln2871*)), *ARX (*p.(Arg483Serfs*46)), *METTL4* (p.(Arg84*)), *IL1RAPL1* (intragenic 0.29 Mb deletion) in five families and a recurrent mutation in *GNE* (p.(Tyr156His)) in one family.

In MRID137a, a variant in GNE was co-segregating with disease phenotype (Fig. 2). *GNE* encodes for a bifunctional enzyme, UDP-N-acetylglucosamine 2-epimerase/*N*-acetyl mannosamine kinase that catalyzes a rate-limiting step in sialic acid biosynthesis. A mutation in this gene has already been reported to cause ARID in another Pakistani family [12]. The variant we identified, p.(Tyr156His), is already reported to cause Hereditary Inclusion Body Myopathy (HIBM) [22]. The clinical features indicative of HIBM, such as muscle weakness or atrophy, were not present in our family. The clinical feature of seizures in our family is suggestive of sialuria which is an autosomal dominant disorder. Harripaul et al., 2018 and our finding anticipate previously unreported autosomal recessive form of sialuria with ID as an additional clinical phenotype. Moreover our finding adds into the phenotypic variability of the p.(Tyr156His) *GNE* mutation.

MRID137b family has a missense variant, p.(Gly117Ala), in *CCS* which encodes a copper chaperon of superoxide dismutase. CCS is a 274 amino acid long cytosolic protein that is involved in delivery of copper to SOD1 via protein–protein interaction mediated by domain II, a highly homologous (SOD1 like) domain of CCS [23, 24]. A missense mutation, p.Arg163Trp, in *CCS* domain II along with a homozygous mutation (p.Tyr366*) in *SLC33A1* gene is reported to cause severe muscular hypotonia, hypoglycemia, pericardial effusion, developmental regression, epilepsy, congenital cataracts, bilateral hearing loss, developmental delay, cerebral palsy and very low serum Cu and ceruloplasmin levels in a Turkish patient [25]. Clinical phenotype such as muscular hypotonia, seizures, anemia and speech impairment are overlapping between Turkish patient and MRID137-6. It is presumed that clinical features of congenital cataract, hearing loss and developmental regression in Turkish patient were attributed to a nonsense mutation in *SLC33A1* gene [25]. The ceruloplasmin levels in our patients are normal (33.2 mg/dL Reference: 16-45 mg/dL) but reduced in the Turkish patient. The ceruloplasmin secretion is reduced in patients with *SLC33A1* mutations [26], therefore we can conclude that probably *CCS* mutation alone has no effect on ceruloplasmin level in our patient. Immunoprecipitation studies showed that the p.Arg163Trp mutation in CCS disrupts its binding affinity to SOD1 [25]. The variant identified in MRID137b is also localized in domain II of CCS protein which may lower the binding capacity to SOD1, probably resulting in the disease phenotype in this family.

Family MRID143 and the X-linked MRID149 carried nonsense and splice site variant in *C5orf42* and *ARX* gene respectively. *C5orf42*, also known as *CPLANE1,* encodes a cilia transition zone protein which plays an important role in ciliogenesis and mitotic progression [27, 28]. Disease causing mutations in this gene are known to cause Joubert syndrome 17 and Oral-Facial-Digital (OFD) syndrome VI [16, 17]. The presence of strabismus (Table 1) in patients of MRID143 family supports the involvement of *C5orf42* gene. The protein encoded by *ARX* (Aristaless-related homeobox) gene plays a pivotal role in tangential migration and differentiation of GABAergic and cholinergic neurons [29, 30]. Disease causing mutations in *ARX* are known to causes ID with or without ASD.

The second X-Linked family, MRID165, harbored deletion in *IL1RAPL1* (Interleukin 1 Receptor Accessory Protein Like 1) gene which is known to cause ID with or without co-morbid features. IL1RAPL1 regulates dendritic synapse formation and stabilization [31–34]. Intragenic deletions of *IL1RAPL1* have been identified in ID patients. Deletion of exon 6 was reported in two families with moderate ID, language delay, facial dysmorphism, autistic and aggressive behavior. Δex6 functional analysis revealed that the mutant IL1RAPL1 is unstable and mislocalized within dendrites [31]. Deletion of exon 3–7 has been reported to cause autistic behavior and ID [35], deletion of exon 2–5 has been reported in ID with hyperactivity [36], deletion of exon 3–5 has been reported to cause microcephaly, dysmorphic features and ID [37, 38]. *IL1RAPL1* pericentric inversions characterized in ID affected patients suggest that this region is prone to non-homologous recombination events [39–41]. In our family (MRID165), deletion of exon 6 and flanking intronic regions is associated with moderate ID, poor speech development and impaired cognition, but all three patients have normal head circumference. Additionally, we could not identify any difference in the phenotypic presentation of male (hemizygous for *IL1RAP1* deletion) and female (heterozygous for *IL1RAP1* deletion) individuals of this family (Fig. 1). Phenotypic expression in carrier XLID females has been reported in previous studies and almost 13 genes are known to give equivalent or more expression in carrier females [42]. A de novo frameshift mutation, p.Ile367Serfs*6, in *IL1RAPL1* has been reported in a girl with ASD [35]. It is suspected that the functional consequence of the deletion in our family will be same as reported earlier [31]. We anticipate that ID phenotype in the female patient, heterozygous for IL1RAPL1 deletion, is probably due to X chromosome inactivation.

MRID170 carried a nonsense variant in *METTL4* genes which co-segregate with ID phenotype (Fig. 2). *METTL4* is a member of MT-A70 domain containing adenine methyltransferases [43]. It performs the function of N6-methyladenine (6 mA) modification in DNA which is responsible for epigenetic silencing [44, 45]. *Mettl4* KO mice studies showed anatomical defects including craniofacial dysmorphism and anophthalmia in KO mice as compared to wild type controls [46]. A disease-causing nonsense variant, p.(Cys196*), in this gene has already been identified to cause ARID in a family with Pakistani descent [13]. Furthermore, mutations in other methyltransferase-like gene, *METTL5* and *METTL23*, have also been implicated in ID [47, 48].

Family MRID175 has three rare variants including a frameshift variant in *C7orf50*, and missense variants in *ABCB5* and *ELFN1* genes. Extracellular leucine rich repeat and fibronectin Type III domain containing 1 (*ELFN1*) expression is evident in a subset of interneurons in cortex and hippocampus and is localized mostly to excitatory post-synaptic sites where it acts as a trans-regulator of mGluR7 [19, 49]. *Efln1* knock out (KO) mice exhibits seizures and hyperactivity, and similar neurological phenotype was observed in mGluR7 deficient mice suggesting a close functional relationship between the two proteins [19, 50, 51]. Heterozygous missense variants, p.Ala481Val, p.Arg650Cys, p.Asp678Asn, p.Arg691Trp, in *ELFN1* have been identified in a Japanese cohort diagnosed with epilepsy, autism spectrum disorder (ASD), and attention deficit hyperactivity disorder [19]. Affected individuals of MRID175 family are homozygous for missense variant c.149C>A (p.(Pro50His)) in *ELFN1* gene and presented moderate ID, poor speech and cognition deficit. A 16-year-old female individual from MRID175 currently suffers from epilepsy, but it was absent in two younger individuals of this family. Considering the earlier reports of *ELFN1* involvement in neurological disorders in heterozygous state, we can conclude that homozygous variant detected in MRID175 is a likely cause of moderate ID.

## Conclusion

This study supports the genetic and phenotypic heterogeneity of Mendelian forms of ID. We identified novel variants in previously reported ID causing genes i.e. *ARX, C5orf42* and *METTL4,* a recurrent variant in GNE which is involved in causing ID and a CNV in *IL1RAPL1*. We also report novel missense variants in *ELFN1* and *CCS* to be implicated in intellectual disability which are not reported previously in ID phenotype.

## Supplementary Information

**Additional file 1: Fig. S1.** Raw agarose gel electrophoresis image showing the results of IL1RAPL1 Deletion mapping in MRID165. D1 represents the PCR product (~ 2000 bp) obtained with primers flanking the deleted region while D2 represents PCR product obtained with primers located within the deleted region. Individual showing only D1 band (IV-4, IV-5) are hemizygous for deletion and individuals with D2 band (III-1, IV-3) are homozygous for normal allele. Individuals (III-2, IV-6) with both bands are heterozygous for deletion.

## Data Availability

Written consent forms of the participating subjects or their legal representatives, are available upon request. The raw whole-exome sequencing data are not publicly available due to privacy or ethical restrictions. Genotype and DNA sequencing data generated or analyzed within this study are available upon an email request from the corresponding author Muhammad Ansar.

## Abbreviations

ID: Intellectual disability
SNP: Single nucleotide polymorphism
CCS: Copper chaperon of superoxide dismutase
SOD1: Superoxide dismutase 1
ELFN1: Extracellular leucine rich repeat and fibronectin Type III domain containing 1
GNE: UDP-*N*-acetylglucosamine 2-epimerase/*N*-acetyl mannosamine kinase
ABCB5: ATP-Binding Cassette, Subfamily B, Member 5
ARX: Aristaless-related homeobox
*CPLANE1*: Ciliogenesis and Planar Polarity Effector 1
*IL1RAPL1*: Interleukin 1 Receptor Accessory Protein Like 1
METTL4: Methyltransferase-Like 4
*METTL5*: Methyltransferase-Like 5
*METTL23*: Methyltransferase-Like 23
OFD: Oral-Facial-Digital Syndrome
mGluR7: Metabotropic Glutamate Receptor 7
6 mA: N6-methyladenine
HIBM: Hereditary Inclusion Body Myopathy
ASD: Autism Spectrum Disorder
LRR: Leucine rich repeat
CNV: Copy Number Variant
NGS: Next Generation Sequencing
DNA: Deoxyribonucleic acid
HBD: Homozygous by descent
PCR: Polymerase chain reaction
WES: Whole exome sequencing
gnomAD: Genome aggregation database
