# Supplementary figures and images for: Exome sequencing identifies novel and known mutations in families with intellectual disability
Source: BMC Med Genomics. 2021 Aug 27;14:211. doi: 10.1186/s12920-021-01066-y (PMC8399827; doi:10.1186/s12920-021-01066-y)

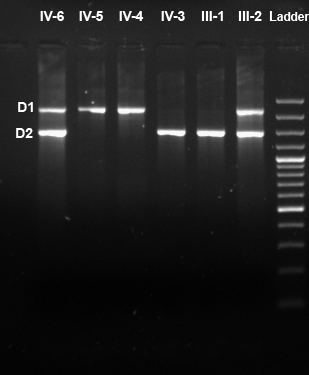

Supplement: Supplementary file 1 — Additional file 1: Fig. S1. Raw agarose gel electrophoresis image showing the results of IL1RAPL1 Deletion mapping in MRID165. D1 represents the PCR product (~ 2000 bp) obtained with primers flanking the deleted region while D2 represents PCR product obtained with primers located within the deleted region. Individual showing only D1 band (IV-4, IV-5) are hemizygous for deletion and individuals with D2 band (III-1, IV-3) are homozygous for normal allele. Individuals (III-2, IV-6) with both bands are heterozygous for deletion. [file 12920_2021_1066_MOESM1_ESM.jpg]
